# Data assimilation and multisource decision-making in systems biology based on unobtrusive Internet-of-Things devices

**DOI:** 10.1186/s12938-018-0574-5

**Published:** 2018-11-06

**Authors:** Wei-Hua Tang, Wen-Hsien Ho, Yenming J. Chen

**Affiliations:** 1 Division of Cardiology, Department of Internal Medicine, National Yang-Ming University Hospital, Yilan, Taiwan; 2 Department of Healthcare Administration and Medical Informatics, Kaohsiung Medical University, Kaohsiung, Taiwan; 3 Department of Medical Research, Kaohsiung Medical University Hospital, Kaohsiung, Taiwan; 4 Department of Logistics Management, National Kaohsiung University of Science and Technology, Kaohsiung, Taiwan

**Keywords:** Multisource evidence, Data assimilation, Systems biology, Pervasive sensing, Bayesian network, Machine learning

## Abstract

Biological and medical diagnoses depend on high-quality measurements. A wearable device based on Internet of Things (IoT) must be unobtrusive to the human body to encourage users to accept continuous monitoring. However, unobtrusive IoT devices are usually of low quality and unreliable because of the limitation of technology progress that has slowed down at high peak. Therefore, advanced inference techniques must be developed to address the limitations of IoT devices. This review proposes that IoT technology in biological and medical applications should be based on a new data assimilation process that fuses multiple data scales from several sources to provide diagnoses. Moreover, the required technologies are ready to support the desired disease diagnosis levels, such as hypothesis test, multiple evidence fusion, machine learning, data assimilation, and systems biology. Furthermore, cross-disciplinary integration has emerged with advancements in IoT. For example, the multiscale modeling of systems biology from proteins and cells to organs integrates current developments in biology, medicine, mathematics, engineering, artificial intelligence, and semiconductor technologies. Based on the monitoring objectives of IoT devices, researchers have gradually developed ambulant, wearable, noninvasive, unobtrusive, low-cost, and pervasive monitoring devices with data assimilation methods that can overcome the limitations of devices in terms of quality measurement. In the future, the novel features of data assimilation in systems biology and ubiquitous sensory development can describe patients’ physical conditions based on few but long-term measurements.

## Background

Bioinformatics, together with machine learning, can extend the scope of medical or biological practices. Biological and medical diagnoses and treatments sometimes need appropriate instruments. Machine-assisted medical diagnosis focuses on automatic disease inference from observed symptoms. However, physical laws are limited. Therefore, advanced inference technologies must be developed to complement device measurements. In contrast to human experts without knowledge of specific diagnostic criteria, statistical inference avoids type I and II errors. However, the assistive role of a machine must be consistent, and the final diagnostic decision must be ordered by a physician. As a consequence, full automation provides insufficient benefits in practical applications.

Current clinical diagnoses often provide decisions by comparing physiological data with several heuristically defined thresholds; for example, “infarction is true if ST-waves elevation exceeds 60%”. However, this scheme is only good for a human expert but not for a dummy machine. A trained doctor can consolidate all necessary data and replace relevant numbers with intuitive information to finalize his diagnosis, but a machine cannot perform such replacements. Computerized diagnosis aims to replace human intuition with various comprehensive algorithms and complicated criteria. Nevertheless, replacement has yet to be achieved. Thus, ideal computerized assistance should assess the statistical significance of conclusions and extend the scope of human experts to perform time-consuming and large-quantity investigations, as well as not mimic human processes. Technological advancements have improved backward and forward inferences to provide novel evidence for quality judgment by human experts [1]. New statistical inference methods help doctors observe details that were previously difficult to observe because of high sampling costs in body reaction, such as in blood, radiative, or invasive tests. Moreover, existing technologies have been continuously developed to support high levels of disease diagnoses, such as hypothesis testing, multiple evidence fusion, machine learning, data assimilation, and systems biology.

A highly efficient tool is necessary to combine data from various sources. Data assimilation refers to the quantitative methods which combine observations of variables with system behaviors to estimate internal states and key parameters. In scientific applications, data assimilation is similar to solving an inverse problem with ill-posed condition complex solutions. Although the concept is based on geosciences, data assimilation is a well-developed discipline in various fields, including ocean forecasting [2], paleoclimatology [3], and gene networking [4]. Moreover, data assimilation has been applied to initiate large weather projects, such as the Regional Ocean Modeling System [5].

Current medical research trends include personalized medicine [6] and patient-specific modeling [7]. Influential applications have emerged because of the prominence of Internet of Things (IoT) and progress of cross-disciplinary integration. For example, the multiscale modeling of systems biology from proteins, cells, organs, and individuals integrates the contemporary development of biology, medicine, mathematics, engineering, artificial intelligence, and semiconductor technologies. IoT is a computing concept envisioned as the physical objects connected to the Internet with the ability to identify themselves to other devices [8–10]. Researchers have gradually designed ambulant, wearable, noninvasive, unobtrusive, low-cost, and pervasive monitoring devices based on IoT-related monitoring devices. Moreover, with the successful integration of cross-disciplinary research, personalized medicine has become considerably realistic. Additional data from ambulant devices should be provided to obtain novel evidence. Vital signs are critical to diagnosis, but some signs are difficult to obtain [11]. Ideal transducers should be based on temporal and spatial patterns and physical signals, such as acoustic, electrical, and optical measurements [12, 13]. New generations of automatic diagnostic systems should allow personalized treatments. In the future, novel data assimilation methods for systems biology and ubiquitous sensory can reveal a patient’s physical responses, which are modeled as a series of inverse inferences in each scale of system models [4, 14, 15].

Two problems are encountered in cross-disciplinary research. First, not all wearable devices can easily collect useful vital signs. Second, accurate treatments suggestion becomes challenging from mutually conflict information even if the first problem can be solved. Backward inferences can be achieved through Bayesian techniques, because inverse problems attributed to multiscale modeling are ill-posed. Hidden states, cause-effect relations, missing measurement values, and control consequences are obtained by solving inverse problems. Thus, a potential treatment can be derived when all internal states are clarified [16, 17].

Similar to those portrayed in science fiction movies, instruments have been developed to observe a human body from multiple perspectives, including cell and organ shapes and the interaction timing of objects, such as a car technician running a computerized diagnosis on the timing of an engine ignition sequence. Using this tool, doctors can easily perform an immediate diagnosis. Before this scope was invented, mathematical inference was necessary to overcome measurement inaccuracy. Hence, inverse problems should be solved with advanced data assimilation techniques to provide new data evidence.

**Fig. 1:**
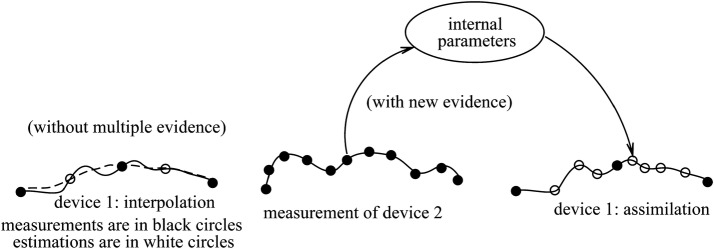
Data assimilation based on multiple evidence can enhance measurement accuracy

**Fig. 2:**
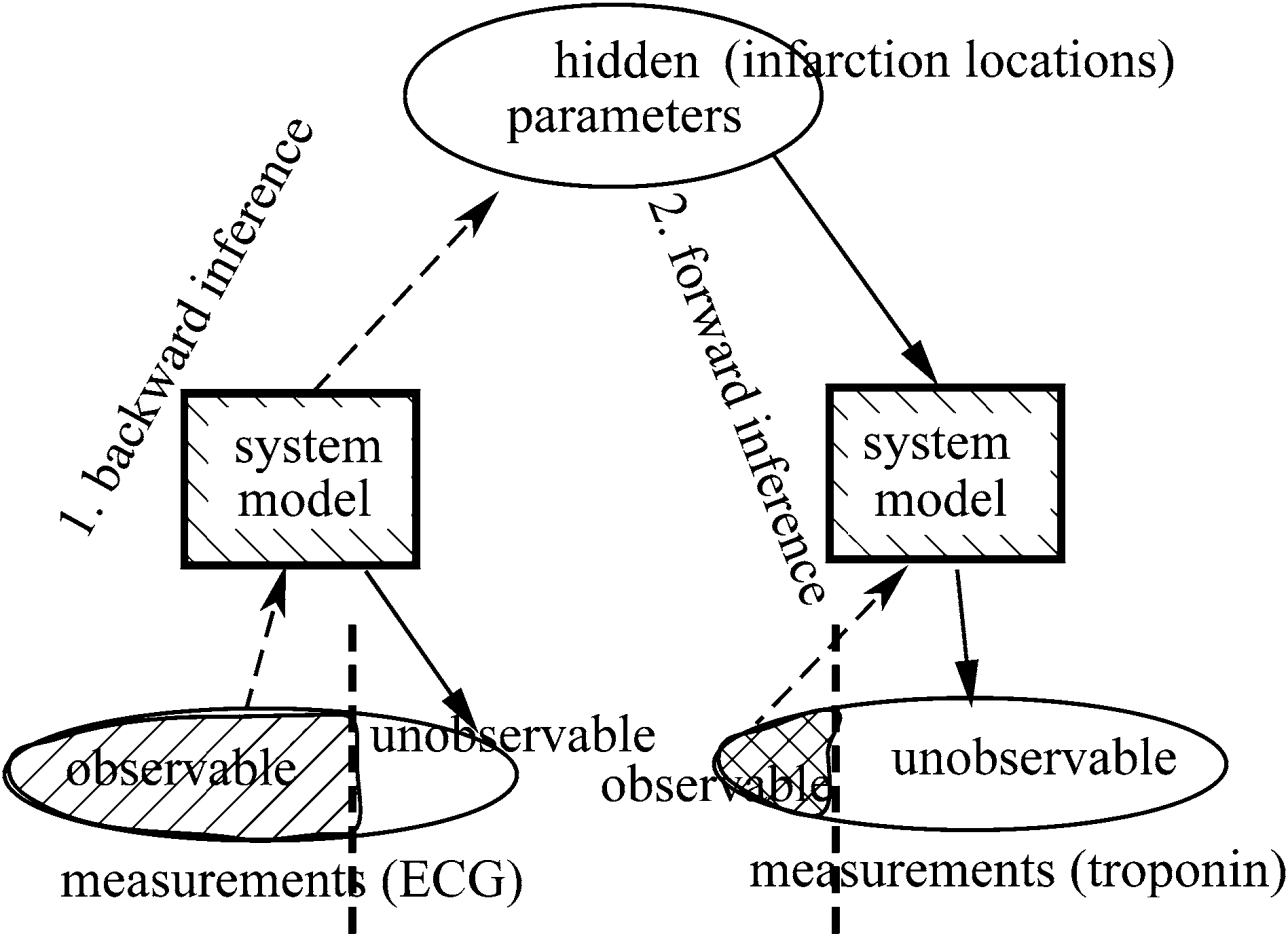
Concept of data assimilation

In the left panel of Fig. 1, the estimation of the unmeasured signals is inaccurate when one measuring device is used. The true signal should be in a solid line, and the estimated signal become the dashed line, because the estimation algorithm is insufficient. In this case, the consequence of estimation is equivalent to that of interpolation. Using data assimilation algorithm (right panel of Fig. 1), the estimation becomes accurate and adds new information to Device 1 by introducing Device 2. For example, a blood test cannot be performed frequently, but non-invasive galvanic potential can help improve the estimation of other measurements. In Fig. 2, new evidence can be derived through observations of common causes. Through known system models, one observable can derive hidden parameters and then derive other unobservable. For example, from ECG we can estimate infarction locations and then we can estimate troponin concentration without physical measurement.

**Fig. 3:**
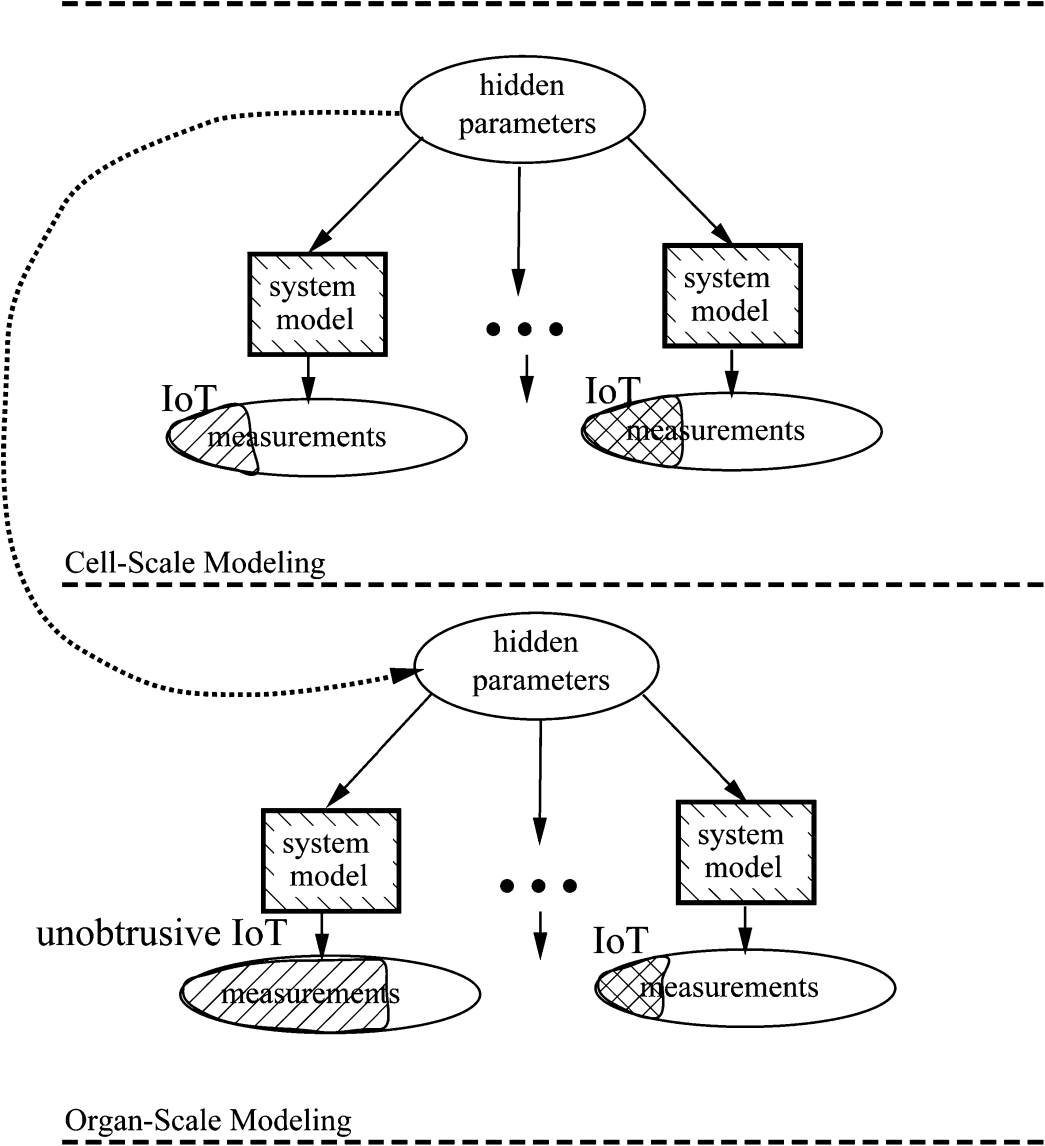
Multiscale data assimilation based on Internet of Things devices

Identifying the relationship between causes and effects is important in assimilation. For example, the relationship could be a linear combination of several causative factors or autoregressive functions in several change rates. Such relationships are described on three levels: interaction, constraint, and mechanism-based models [18]. We consider the mechanism-based model, referred to as the relation system model, that serves as the key to performing correct forward and reverse inferences. When integrated with cross-disciplinary knowledge, IoT-based multiscale data assimilation based (Fig. 3) can achieve the goals of personalized medicine. The accuracy of estimation can be mutually compensated between different scales.

Data assimilation is implicated in personalized medicine when genome and proteome data cannot be read at home. To our knowledge, efficient and unobtrusive IoT devices that are accessible and can process multisource decision-making for data assimilation have yet to be developed. However, commercially available prototypes have been designed. Cardiovascular system-related data, such as heart rate (HR) regularity and blood pressure (BP), can be recorded, monitored, and analyzed in a cloud control center with advanced mobile devices, such as mobile phones, smart watches, patient monitoring devices, or personal digital assistants [19]. Moreover, the diagnosis of cardiac arrhythmias, such as atrial fibrillation, can be easily made with such devices; subsequently, early disease management shall be applied, and the undesirable complications of diseases, such as stroke, may be prevented [20]. For a patient with an intracardiac electronic device, such as a pacemaker or a defibrillator, a home remote care system can be applied to upload cardiac electrophysiological information and body fluid status. An increase in tissue impedance, which may indicate fluid retention and heart failure, can be detected early, and doctors can be immediately informed via the system. Moreover, early diuretic administration and intervention can ameliorate heart failure, avoid hospitalization and respiratory failure, and minimize the economic burden on governments [21, 22]. Furthermore, implantable continuous glucose monitoring devices are available commercially. Glucose sensors can wirelessly communicate with an external receiver, such as a smartphone, and offer a high-bandwidth data source for a health provider; and these sensors are applicable to new diabetes-related health information technology applications [23–25].

## Data collection devices for bioinformatics

Sensory devices are key enablers of a new data assimilation paradigm. To obtain ambulant, wearable, noninvasive, unobtrusive, low-cost, and pervasive monitoring devices, researchers have extensively investigated various measurements with single devices and integrating numerous devices. Advancements in hardware technology and mathematical analysis have continuously expanded the field of sensory devices.

The importance of pervasive computing and IoT technology has been promoted in healthcare applications [26, 27]. The role of wearable devices in the paradigm of p-health, namely, participation, prevention, prediction, preemptive, pervasive, and personalized, has been highlighted [28]. With various technologies essential for patient monitoring, wireless devices and microchips contribute to the success of future applications [12, 29, 30]. In order to monitor chronic diseases and perform preventive care, pervasive computing is necessary for pursuing the acceptance of patients [13, 31]. A support vector machine has been proposed that combines measurements from health professionals with continuous data acquired from wearable devices to predict patient deterioration that may readmit to Intensive Care Units (ICU) [32]. Personalized data and healthcare information, along with cloud computing, have been transmitted and processed over the cloud [33].

Among the requirements of a wearable monitoring device for common vital signs, such as electrocardiogram (ECG), ballistocardiogram (BCG), HR, respiration rate (RR), BP, blood oxygen saturation (SpO_2_), core/surface body temperature, posture, and physical activities, unobtrusiveness is probably the most important property for continuous monitoring [34]. Several methods can be unobtrusive to patients [34], including capacitance-coupled sensing for ECG, electroencephalogram, (EEG) and electromyogram (EMG); photoplethysmographic (PPG) sensing for SpO_2_, HR, RR, and BP; pulse wave propagation sensing for BP; piezoelectric sensing for respiration, heart sound, and BCG; inductive plethysmogram and optical fibers into textiles for respiration; and radar sensing for lung or heart motions. With the physical capability of individual sensors, multiple sensors can be deployed to acquire the same vital sign. In this case, an effective algorithm in data fusion can resolve the limitations of wearable devices [34].

Sound is apparently the easiest parameter to obtain among commonly measured physiology data [35]. However, this parameter also suffers from contamination by cross-talk, noise, and artifacts. Lung motion involves low- and high-frequency vibrations. Moreover, the air resonance caused by high-frequency components creates an audible sound and can be analyzed to determine the ectopic functioning of lungs. Time–frequency analysis has been performed to extract the wheeze inside normal and abnormal breath sounds [35, 36]. Pneumonia has been automatically detected in breath sounds via short-time Fourier transform and machine intelligence [37]. Abnormal respiration sounds have been detected by tracking instantaneous frequencies, which can be similarly obtained by previous approaches, and envelop, which can be obtained from ensemble empirical mode decomposition [38]. Sound signals from the heart are complicated. Aortic and pulmonary components of the second heart sound have been separated by establishing a nonlinear model of the observed chirp signals [39].

Like sound, light, photographic signals are easily obtainable. For example, PPG is used to derive heart and lung activities using reflection from incident lights; that is, PPG is applied to obtain the heart and respiratory rates independently, and the respiratory rate is subsequently recalibrated with the relationship between the two rates [40]. The accuracy of estimation is commonly cross-verified with these two parameters. In another proposed method [41], rough PPG signals are calibrated with ECG signals.

Electrical signals are important in noninvasive and unobtrusive sensing methods. However, most low-cost devices suffer from noise problems. Various measuring agents, such as current, impedance [42], and capacitance, suffer from low signal-to-noise (S/N) ratio. Heart-correlated impedance changes in the legs are measured for pulse rate, and a comparative result has been obtained under well-controlled conditions [43]. The bathroom weighing scale concept has been applied and an unobtrusive impedance monitor has been developed for cardiovascular health [44]. Moreover, an ECG sensor in wireless hardware has been utilized for long-term wear [45]; as well as current heart monitoring systems [46]. Furthermore, impedance and capacitance have been proposed as potential candidates for long-term wear where measuring electrical potential is unreliable and active motion artifacts have been compensated during surface capacitance measurement [47]. With the prevalence of wearable devices, commercialization has pressured this category of sensors to viability.

Certain organ motions, such as heartbeats or lung aspirations, are easily detected by Doppler radars. However, measuring BP without a cuff is still under development. For example, BP is estimated with pulse wave velocity and transit time, which are considered vital signs [48]. Similarly, BCG and plastic optical fiber technology have collected corresponding pulse waves with unobtrusive cardiovascular monitoring systems [49, 50].

Among available wearable devices, a continuous blood sugar monitor is probably the most wanted functionality; nevertheless, the following unfavorable gaps have yet to be overcome. Personalized wearable devices have been proposed [51], but blood sugar measurement remains challenging [24]. Current technologies for non-invasive glucose monitoring have been reviewed, and results have revealed current drawbacks and potential alternatives [52]. Therefore, breath gas contents and microwave skin reflection have been recommended as alternatives for blood glucose estimation [53, 54]. Moreover, Bayesian methods have been applied to overcome the inaccuracy of plasma glucose measurement from interstitial observations [55].

Data from various devices are irrelevant without a sophisticated medical decision system. A computerized program has been established based on diagnostic assistance [56, 57]. Advanced statistics and medical knowledge have been accumulated throughout technological development [58, 59]. Similar statistics has been shared with systems biology [60]. Medical decision systems at two instances have been reviewed, and several support systems for major clinical decisions have been designed [61, 62]. A model has been implemented based on a diagnostic system to statistically analyze the composition profile of lipoprotein subclasses from clinical chemistry data, which helps predict metabolic disorders and cardiovascular risk [63].

## Fusion of multisource evidence in systems biology

Advanced statistical techniques are necessary to fuse data from multiple sources. A data fusion-based risk assessment framework for human health was proposed in accordance with Dempster–Shafer theory and systems biology principles to achieve excellent evidence fusion [64]. The same evidence fusion theory has been applied to design and fuse multisensory evidence for engine fault diagnosis [65]. A framework with multiscale processes and multiple model choices, such as ordinary or partial differential equations, has been recommended for biological applications [66].

Prediction functions well with the correct system model. Among the most common and traditional models are linear regression and linear time series types. However, linear and time-invariant systems are part of a small portion of the actual world phenomena. Moreover, most systems change with time, and outputs are not linearly proportional to inputs. Some systems can be easily discussed with a simple transformation. For instance, neural network transformation has been implemented to bypass nonlinear problems and perform effective inferences based on extracted features [67, 68]. Models and methods in discovering biomarkers have been reviewed to predict clinical outcomes in the cardiovascular field [69]. Mathematical modeling has been applied to predict atherosclerosis [70].

Systems biology can be easily merged with data assimilation in bioinformatics. For example, the same hierarchical Bayesian estimator with data assimilation has been used to rank several biochemical system models on the basis of hidden parameters [71]. The properties and solutions of inverse problems have been reviewed in terms of systems biology in relation to mathematical modeling and experiments [18]. In systems biology, cell functions should be elucidated in terms of system dynamics [72]. Therefore, the goals of systems biology are consistent with those of data assimilation. A Kalman filter, which is a specific data assimilation method, has been used to recursively estimate hidden parameters in a biological system [73]. The roles of systems biology in predictive and preventative medicine have been highlighted [74, 75]. In the proposed concept of systems medicine, medical genomics is merged with healthcare applications [76]. Moreover, personalized medicine should be based on systems biology [77, 78]. With advances in scientific knowledge, personalized medicine with patient-specific modeling has been achieved. Research progresses on patient-specific modeling have been reviewed, and results have revealed that simulation should address challenges in the dynamics of systems biology instead of solely on 3D image presentation [7].

Bayesian statistics has been improved to support statistical inference in systems biology. Inference methods have been proposed for medical prediction [79]. Computation issues in biology have been determined, and the importance of statistical inference has been explained [60]. A Bayesian network has been constructed without comprehensive observations [80]. Moreover, Bayesian methods, such as biological sequence, microarray, and protein interaction analysis, have been applied to bioinformatics [81]. Future applications, such as statistical inference based on the system information of differential equations, have been proposed through Bayesian methods in bioinformatics [81]. A tutorial on Bayesian network basics in the health domain has been provided [82]. Complementary to computations per se, a multiscale design at protein, cellular, tissue, and organ levels has been recommended [83].

In advanced Bayesian applications, a partially collapsed Gibbs sampler is used in ECG signals to delineate P- and T-waves, which are difficult to locate without using maximum posterior probability [16]. The precision of the parameters in a Bayesian network will not significantly alter the correctness of diagnosis [84]. Moreover, a Bayesian network decision model has been proposed to diagnose dementia-related diseases [85]. Furthermore, a hierarchical Bayesian method has been employed to estimate the posterior distribution of transmural ECG imaging without acquiring accurate geometric parameters [17].

## Data assimilation in biology and medicine

Unobservable data can be readily estimated by Bayesian methods, forward system equations, and few observations, even without detailed parameters, which are then gradually estimated through assimilation. Moreover, the technique applies sparsely observed information to estimate system internal states by acquiring assumptions based on system behaviors typically governed by a set of predefined spatio-temporal equations. Assimilation application can significantly reduce the requirement of measurement [16, 17].

Although well-developed diagnostic decision support systems have been established for general usage, accumulated experiences are helpful for more elaborate applications upon establishing the forward path of system dynamics. New algorithms are necessary for fusing data when observations are collected from different models of system dynamics [32]. Scientific knowledge and applications are important in the success of related developments because system dynamics modeling is crucial in data assimilation. However, a simple linear regression between causes and effects is insufficient for biological and medicinal applications.

Chemical measurements produce the minimum amount of measured data for a required level of accuracy because laboratory experiments are costly and time-consuming [63]. Chemical data assimilation has been reviewed by introducing a computational model in marine biogeochemistry, which combines observed data and system behavior models [86]. Moreover, a Bayesian method and prior knowledge in parameters have been applied to estimate bacterial growth rate using minimum required measurement data in bacterial counting [87].

In data assimilation, automatic diagnosis is performed to achieve disease inference and measurement data completion via a forward system with inferred disease states. The fundamental Bayesian statistics used in data assimilation, including ensemble Kalman filtering, Markov chain Monte Carlo sampling, and hierarchical Bayesian modeling, have been described [88]. Furthermore, stochastic processes in data assimilation that infer high-quality data measurements using frequent low-quality observations and rare accurate observations have been reviewed [89].

Data assimilation mostly focuses on sequential problems; hence, a tractable algorithm is essential for computational feasibility. A relevant sampling of the Monte Carlo method or particle filters have been applied to approximate Bayesian updating within the large space of posterior probabilities [90]. Sequential data assimilation algorithm has been performed based on limited observations for continuous monitoring of soil moisture and temperature [91]. State-space and parameter estimations have been conducted simultaneously using an ensemble Kalman filter [92].

Data assimilation has been applied to resolve various issues in medical applications. A tissue contractility problem has been investigated to predict infarctions by using a sequential data assimilation approach based on a biomechanical heart model established from magnetic resonance imaging data [93, 94]. The myocardial mechanical property has been estimated on the basis of end-diastolic displacement measurements via a data assimilation framework [14]. Moreover, cardiac contraction and relaxation have been determined via a variational data assimilation approach based on a new dynamic model using cine-magnetic resonance imaging (MRI) sequences [15].

In bioinformatics, Bayesian and Markov approaches have been used extensively, but applications that estimate sequentially changing dynamics have yet to be fully developed. Regarding the genomic sequencing problem, semidefinite programming and kernel methods have been used to fuse various types of experimental data using similarities between pairs of genes and proteins [95]. State-space data assimilation with a high moment ensemble particle filter in a sequentially changed gene regulatory network has been performed to estimate hidden state variables, which are inferred from time-course observation data from several information sources [4].

The purposes of medical data assimilation and medical decision support system are different. Data assimilation may create millions of systems for different patients and can be applied to collect personal data, establish models, and obtain new evidence. Moreover, it seldom applies the logic that most people act in such manner that others may follow the same way.

## Conclusions and future development

This review illustrates that IoT technology in biological and medical applications has remarkably influenced next-generation healthcare. However, advanced data processing technologies and cross-disciplinary integration must be developed to complement the physical limitations of portable devices. Relevant technologies are mostly ready to support the desired level of disease diagnosis, such as hypothesis testing, multiple evidence fusion, machine learning, data assimilation, and systems biology. The multiscale modeling of systems biology at protein, cellular, and organ levels has integrated the contemporary development of biology, medicine, mathematics, engineering, artificial intelligence, and semiconductor technologies.

New data assimilation processes enable the fusion of multiple data scales from multiple sources and is, therefore, sufficient to provide the desired precision for diagnosis or medical decisions. Based on monitoring objectives related to IoT devices, researchers have designed ambulant, wearable, noninvasive, unobtrusive, low-cost, and pervasive monitoring devices on the assumption that data assimilation methods can address the limitations of such devices in terms of quality measurement.

In future studies, novel data assimilation approaches in systems biology and ubiquitous sensory studies can help elucidate a patient’s physical conditions with few, nonintrusive, and long-term measurements.
